# Prognostic significance of a negative PSMA PET/CT in biochemical recurrence of prostate cancer

**DOI:** 10.1186/s40644-024-00752-1

**Published:** 2024-08-30

**Authors:** Sara Harsini, Patrick Martineau, Sonia Plaha, Heather Saprunoff, Catherine Chen, Julia Bishop, Scott Tyldesley, Don Wilson, François Bénard

**Affiliations:** 1 Department of Molecular Imaging and Therapy, BC Cancer Research Institute, 675 West 10th Ave, Vancouver, BC Canada; 2 Department of Radiology, University of British Columbia, Vancouver, BC Canada; 3 Department of Radiation Oncology, BC Cancer, Vancouver, BC Canada; 4 Department of Surgery, University of British Columbia, Vancouver, BC Canada

**Keywords:** Prostate cancer, Biochemical recurrence, Prostate-specific membrane antigen, PSMA PET/CT

## Abstract

**Background:**

Prostate-specific membrane antigen (PSMA) positron emission tomography/computed tomography (PET/CT) is becoming standard of care for men with biochemical recurrence (BCR) of prostate cancer. The implications of a negative PSMA PET/CT scan in this population remain unclear. This study aims to assess the outcome of patients with BCR post radical prostatectomy (RP) who have negative [^18^F]DCFPyL PET/CT scan at relapse.

**Methods:**

This is a post-hoc subgroup analysis of a prospective non randomized clinical trial. One hundred and one patients (median age, 75 years) with BCR after RP, who tested negative on [^18^F]DCFPyL PET/CT and subsequently either underwent salvage radiotherapy (sRT) with or without androgen deprivation therapy (ADT) or were followed without active treatment, were included. Freedom from progression (FFP) after negative PSMA PET/CT was determined based on follow-up imaging selected as per clinical practice. Uni- and multivariate Cox regression analyses were performed to examine the association of patients' characteristics, tumor-specific variables, and treatment with clinical progression at the last follow-up. FFP at 1-, 2-, and 3-year were reported using Kaplan Meier analysis.

**Results:**

The median PSA level at PET/CT was 0.56 ng/mL (range, 0.4–11.3). Sixty five (64%) patients were followed without receiving further treatment, and 36 (36%) received sRT (18% to the prostate bed only and 18% to the prostate bed and pelvic lymph nodes) within 3 months of the PSMA PET. Seventeen of the sRT patients (17 of 36, 47%) received concomitant androgen deprivation therapy (ADT). Median follow-up was 39 months. Subsequent clinical progression was detected in 21 patients (21%), with 52% in pelvic lymph nodes, 52% in the prostatic fossa, 19% in distant lymph nodes, 14% in lungs, and 10% in bones. The FFP was 95% (95% CI: 91%-99%) at 12 months, 87% (95% CI: 81%-94%) at 24 months, and 79% (95% CI: 71%-88%) at 36 months. Multivariate Cox regression analysis revealed that an initial International Society of Urological Pathology (ISUP) grade 5 was significantly associated with clinical progression at the last follow-up (hazard ratio, 5.1, *P* value, 0.04). Furthermore, the receipt of sRT correlated significantly with lower clinical progression at the last follow-up (hazard ratio, 0.2, *P* value, 0.03), whereas other clinical and tumor-specific parameters did not. Following surveillance-only and sRT, 29% (19 of 65) and 6% (2 of 36) of patients, respectively, showed clinical progression. In the sRT group, no significant difference was observed in FFP between patients who underwent sRT to the prostatic fossa versus those who received sRT to the prostatic fossa and pelvic lymph nodes, although the numbers in these groups were small.

**Conclusions:**

This study suggests that salvage radiotherapy is associated with a decreased or delayed clinical progression in patients with biochemical recurrence following radical prostatectomy who have negative PSMA PET/CT scan results. The analysis also underscores the prognostic significance of the initial ISUP grade, with ISUP grade 5 being associated with worse outcomes.

**Trial registration:**

Registered September 14, 2016; NCT02899312.

## Background

Prostate cancer (PCa) remains a formidable health challenge worldwide, ranking as the second most prevalent cancer and the fifth leading cause of cancer-related mortality among men [1]. Despite advancements in definitive local therapies such as radical prostatectomy (RP) and radiation therapy (RT), a significant fraction of patients, estimated between 20–50%, will develop biochemical recurrence (BCR) depending on the baseline risk group, characterized by rising prostate-specific antigen (PSA) levels in the absence of detectable disease on conventional imaging [2–4]. The optimal management of BCR, particularly in cases of PSA-only recurrence, continues to spark debate, with treatment options including salvage radiation therapy (sRT), androgen deprivation therapy (ADT), combined ADT and sRT, and active surveillance [5–7].

The advent of prostate-specific membrane antigen (PSMA) positron emission tomography/computed tomography (PET/CT) is changing the imaging landscape for PCa. By targeting the PSMA protein, which is overexpressed in the majority of prostate cancer cells, PSMA PET/CT offers superior sensitivity for detecting both local and distant disease spread, facilitating informed treatment decisions [8]. The recent FDA approval of radiotracers such as [^68^Ga]Ga-PSMA-11 and [^18^F]DCFPyl has further advanced our capability to delineate disease extent in patients experiencing BCR [9, 10].

The role of postoperative radiation therapy, with or without ADT, as a curative strategy for patients exhibiting adverse risk features or BCR following RP has been the subject of extensive research. Recent evidence from randomized trials and meta-analyses indicates that early salvage radiotherapy (sRT) at low PSA levels may serve as an effective alternative to adjuvant RT, offering similar oncological outcomes with fewer side effects [11–14]. The implementation of PSMA PET imaging as a standard staging tool for both primary and recurrent PCa has prompted significant shifts in clinical practice. PSMA PET has demonstrated superior lesion detection over conventional imaging techniques in patients with BCR post-RP [15–18]. This leads to a pivotal question: do patients with BCR and negative PSMA PET results also benefit from timely sRT? Despite the European Association of Urology (EAU) endorsing early sRT regardless of PSMA PET results, the evidence from prospective studies is scant, and retrospective data supporting this recommendation are limited [19–21]. Moreover, sRT is not without its complications, which can include urinary incontinence, erectile dysfunction, second malignancy, and bowel dysfunction, among others [22]. Many men facing BCR after RP may prefer to delay sRT to avoid these potential undesirable effects.

While previous studies have investigated the prognostic value of negative PSMA PET/CT scans in biochemical recurrence of prostate cancer, there remains a need for further clarification in this area [23–26]. Therefore, we conducted a retrospective analysis on BCR patients post-RP who exhibited negative [^18^F]DCFPyL PET/CT scans, comparing those who received sRT to those managed conservatively without active treatment. The objective was to provide additional insights into the prognostic value of negative PSMA PET/CT scans in managing BCR following RP. In addition, we aimed to ascertain if sRT subsequent to a negative PSMA PET/CT scan could reduce or delay clinical progression in this patient cohort.

## Methods

### Study design and patient population

This study is a post-hoc subgroup analysis of data from an ongoing prospective non-randomized clinical trial (ClinicalTrials.gov NCT02899312) that enrolled subjects with (1) biochemical recurrence of prostate cancer following RP with a PSA > 0.4 ng/mL and a subsequent increase, (2) BCR after initial curative therapy with RT with a PSA level > 2 ng/mL above the nadir post-therapy, (3) castration-resistant prostate cancer (CRPC) with PSA ≥ 2.0 ng/mL and two consecutive rises above nadir with castrate levels of testosterone (< 1.7 nm/L), and (4) suspicious but inconclusive findings for metastatic disease on other imaging examinations. Exclusion criteria included medical instability, inability to provide written consent, inability to fit through the PET/CT bore (70 cm diameter), inability to lie supine for imaging, exceeding the safe weight of the PET/CT bed (204.5 kg), and ECOG > 2. The clinical trial did not mandate the treatment approach based on the results of the PSMA PET/CT scan, and this was left to the discretion of the participating subjects’ care team. The clinical trial was approved by the UBC/BC Cancer Research Ethics Board and by Health Canada. Written informed consent was obtained from all subjects prior to enrollment in the study.

We retrospectively assessed participants with castration-sensitive disease who met inclusion criterion (1), entered our prospective study between March 2017 and September 2022, and tested negative on PSMA PET imaging (155 out of 1637). Patients who underwent any form of local therapy other than radical prostatectomy prior to PSMA PET/CT were excluded from this analysis (54 out of 155). One hundred and one patients met the above-mentioned criteria and were included in this study.

### Procedures

[^18^F]DCFPyL was synthesized and PET/CT scans were performed according to previously described methods [27, 28]. After fasting for 4 h, participants received an intravenous injection of 237–474 MBq [^18^F]DCFPyL, adjusted for body weight with a 10% variation in target activity. At 120 min post-injection, patients underwent whole-body imaging from the vertex to mid-thigh using a Discovery PET/CT 600 or 690 (GE Healthcare). A non-contrast-enhanced CT scan was acquired for localization and attenuation correction (120 kV, automatic mA selection ranging from 30–200 mA, and a noise index of 20). This was followed by a whole-body PET scan from top of head to proximal femurs, acquired over 2–4 min per bed position, adjusted for participant girth, and reconstructed using the ordered subset expectation maximization algorithm and point-spread function modeling.

### Image interpretation, follow-up and outcome measures

Images were interpreted by experienced nuclear medicine physicians with access to all clinical data on Oasis (Segami), AW Workstation (GE Healthcare) or Hermia (Hermes Medical Solutions). PSMA PET/CT results were provided to the referring clinician, and subsequent imaging follow-ups and management plans were documented for each patient. Post-PSMA PET management was recorded for each patient, including the date and type of treatment initiated (surveillance, systemic, or local therapy). Patients in the current study were either followed without receiving any further active treatment (surveillance group) or underwent sRT within 3 months of their negative PSMA PET/CT result. For those undergoing sRT, treatment was administered to the prostate bed with or without elective pelvic nodal irradiation within 3 months of their negative PSMA PET. Target volume definition, delivered dose, and the use of ADT were at the discretion of the treating physician. The majority of patients received 66 Gy in 33 fractions to the prostate bed, and for those with pelvic RT, 46 Gy in 23 fractions to the nodes in an initial phase using volumetric modulated arc therapy. Radiological follow-up was at the discretion of the subjects’ physician and included repeat [^18^F]DCFPyL PET/CT, [^68^Ga]PSMA-11 PET/CT, MRI, CT, and/or bone scintigraphy, with the interval ranging from 1 to 6 months.

The outcomes recorded were clinical progression and freedom from progression (FFP). Clinical progression was identified through evidence of recurrent disease sites on follow-up imaging. FFP represents the proportion of patients without evidence of disease recurrence at specified time points during follow-up imaging studies. Follow-up time spanned from the negative PSMA PET/CT date to the last documented imaging, with patients not showing clinical progression by the last follow-up considered censored.

### Statistical analysis

Statistical Analysis involved presenting categorical variables as absolute and relative frequencies. Descriptive statistics were expressed as means (± SD) or medians [range] following the Kolmogorov–Smirnov test's distribution assessment. FFP values were estimated using the Kaplan–Meier method, with differences assessed by the log-rank test. Covariates in the univariate Cox regression included initial pathologic T and N stages, ISUP grade, time to biochemical recurrence, PSA metrics, receipt of post-PET/CT sRT, and ADT. Factors with *p* < 0.1 in univariate analyses were included in a multivariate Cox model. Hazard ratios (HR) with 95% confidence intervals not spanning 1 were deemed significant. Two-sided *P* values less than 0.05 were considered statistically significant. Analyses were performed in R (version 4.3.2; The R Foundation for Statistical Computing, General Public License).

## Results

The study included 101 patients with biochemical recurrence of prostate cancer post radical prostatectomy, all of whom tested negative on [^18^F]DCFPyL PSMA PET/CT. The median age was 75 years (range: 55–92 years). The cohort comprised patients who underwent sRT or were followed without initial active treatment after their negative PSMA PET/CT scan. The majority of patients had primary tumors classified as pT2 (46%) and pT3 (49%), with a distribution across ISUP grades, indicating a diverse cohort in terms of tumor aggressiveness and stage at diagnosis. The median PSA level at the time of the PSMA PET/CT scan was 0.6 ng/mL (range: 0.4–11.5 ng/mL). The median follow-up time was 39 months (range: 12–71 months). The cohort's baseline characteristics are summarized in Table 1.

**Table 1:** Patient and treatment characteristics

Characteristic	Entire cohort	Surveillance subgroup	sRT subgroup
Number of patients	101	65	36
Age at PET/CT (years), median (range)	75 (55–92)	76 (62–92)	74 (55–80)
ISUP grade			
1	10 (10%)	10 (15%)	0 (0%)
2	38 (38%)	20 (31%)	18 (50%)
3	32 (32%)	21 (32%)	11 (31%)
4	6 (6%)	3 (5%)	3 (8%)
5	12 (12%)	8 (12%)	4 (11%)
Missing	3 (3%)	3 (5%)	0 (0%)
Primary tumor classification			
pT2	46 (46%)	27 (42%)	19 (53%)
pT3	49 (49%)	32 (49%)	17 (47%)
Missing	6 (6%)	6 (9%)	0 (0%)
Primary nodal status			
pN0	72 (71%)	40 (62%)	32 (89%)
pN1	10 (10%)	8 (12%)	2 (6%)
pNx	14 (14%)	12 (19%)	2 (6%)
Missing	5 (5%)	5 (8%)	0 (0%)
Time from diagnosis to recurrence (years), median (range)	5 (0.5–32)	7 (0.5–32)	2 (0.5–16)
PSA at PET/CT (ng/ml), median (range)	0.6 (0.4–11.3)	0.6 (0.4–11.3)	0.5 (0.4–6.5)
PSA-DT at PET/CT (mo), median (range)	6.9 (-221.2–113.8)	8.2 (-221.2–113.8)	5.9 (-9.6–99.1)
PSA velocity at PET/CT (ng/ml/year), median (range)	0.4 (-838.9–17.5)	0.3 (-106.4–17.5)	0.5 (-838.9–9.6)
Post-PET ADT	17 (17%)	0 (0%)	17 (47%)
Follow-up time (mo) since PET/CT, median (range)	39 (12–71)	42 (12–71)	36.5 (12–68)

*PET/CT* Positron emission tomography/computed tomography, *ISUP* International Society of Urological Pathology, *RP* radical prostatectomy, *RT* radiation therapy, *ADT* Androgen deprivation therapy, *PSA* Prostate-specific antigen, *PSA-DT* Prostate-specific antigen doubling time, *sRT* salvage radiotherapy

Post-PET/CT scan, 36 patients (36%) received sRT within 3 months of their negative PSMA PET/CT results, of which 17 (47%) were also treated with concurrent ADT, while 65 (64%) were followed without further intervention. The type of ADT included luteinizing hormone-releasing hormone (LHRH) agonists (12 patients), anti-androgens (3 patients), and a combination (2 patients). The duration of ADT varied among patients, ranging from 6 to 24 months, with the majority (10 patients) receiving 12 months of therapy. Of the 36 patients in the sRT subgroup, 18 (50%) received sRT exclusively to the prostate bed, while the remainder also underwent elective pelvic nodal irradiation.

Out of the entire cohort, 21 patients (21%) experienced clinical progression. Clinical progression was observed in 29% (19 of 65) of the surveillance-only group, and 6% (2 of 36) of patients in the sRT group. Both failures in the sRT group occurred in patients with ISUP grade 5 tumors. Eleven patients (52%) had clinical relapse within the pelvic lymph nodes, 11 (52%) in the prostatic fossa, 4 (19%) in distant lymph nodes, 3 (14%) in the lungs, and 2 (10%) at skeletal sites, with some patients showing evidence of disease recurrence in multiple sites. Follow-up imaging identified clinical relapse outside the prostatic fossa in 14 patients (14%).

**Fig. 1:**
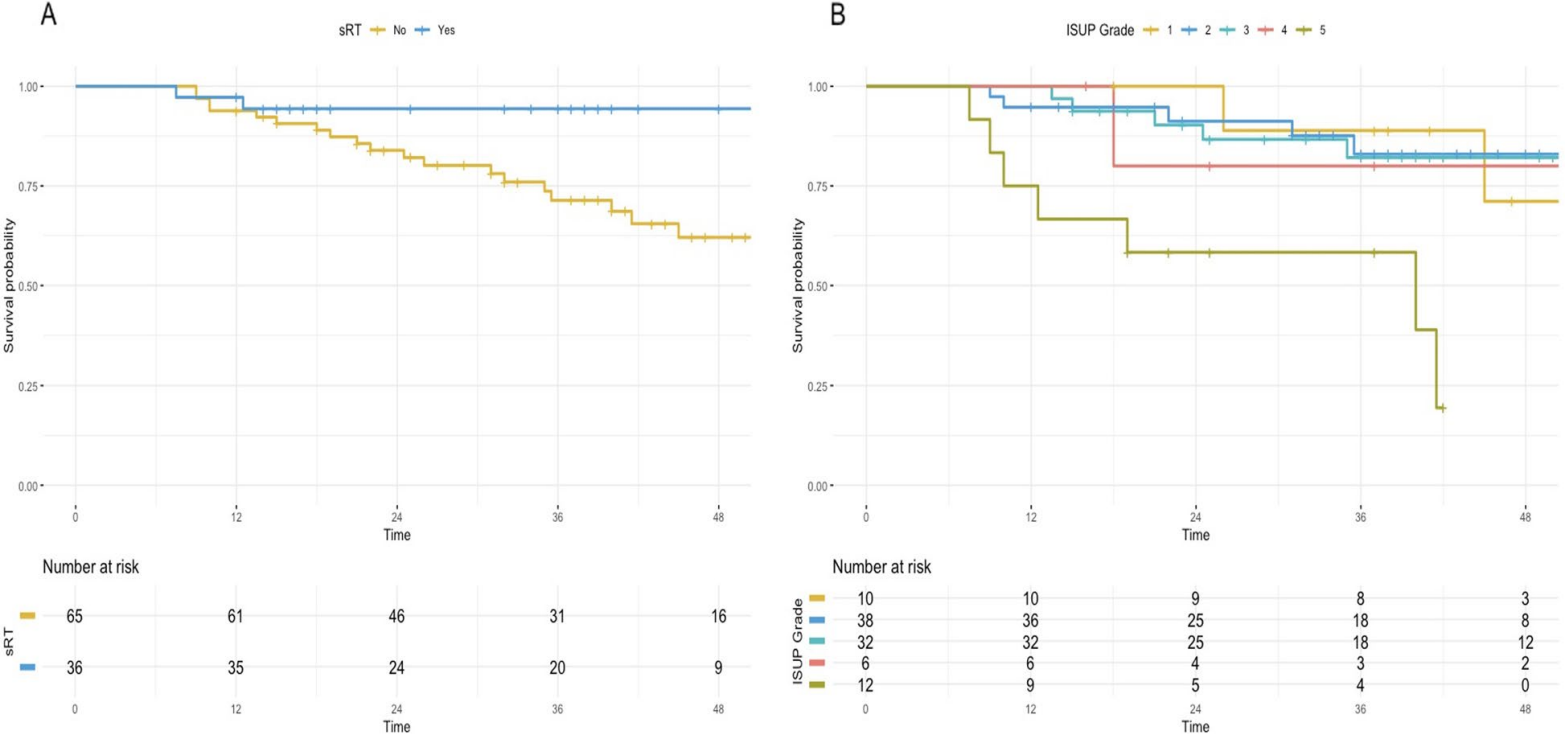
Kaplan–Meier plots comparing the clinical progression-free survival of patients with a prior negative PSMA PET/CT. Subgroup analyses are presented according to: (**A**) receipt of salvage radiotherapy or follow-up with no further treatment; and (**B**) initial ISUP grade (Note: ISUP grade records were unavailable for 3 patients). The curves are truncated when the number at risk falls below 5

Survival analysis revealed that the median clinical progression-free survival (CPFS) was not reached for several subgroups. Kaplan–Meier analysis estimated FFP rates at 95% (95% CI: 91%-99%) at 12 months, 87% (95% CI: 81%-94%) at 24 months, and 79% (95% CI: 71%-88%) at 36 months. For the surveillance group, FFP rates were 94% (95% CI: 88%-100%) at 12 months, 84% (95% CI: 75%-94%) at 24 months, and 71% (95% CI: 60%-85%) at 36 months. For the sRT group, FFP rates were 97% (95% CI: 92%-100%) at 12 months, 94% (95% CI: 87%-100%) at 24 months, and 94% (95% CI: 87%-100%) at 36 months. Notably, the 2-year (p = 0.04) and 3-year (p = 0.02) FFP rates were significantly higher in the sRT group compared to the surveillance group (Fig. 1). No significant difference in FFP was observed between patients who underwent sRT to the prostatic fossa versus those who received sRT to both the prostatic fossa and pelvic lymph nodes. A detailed breakdown of the incidence of FFP by ISUP grade, primary tumor classification, and receipt of post-PET sRT is presented in Table 2.

**Table 2:** Incidence of freedom from progression (FFP) or progressive disease according to different prognostic indicators

	**Subset**	**Subjects**^**a**^	**Progressive disease**^**a**^	**12-month FFP (95% CI)**	**24-month FFP (95% CI)**	**36-month FFP (95% CI)**
All subjects	-	101 (100%)	21 (21%)	95% (91%-99%)	87% (81%-94%)	79% (71%-88%)
ISUP grade	1	10 (10%)	2 (50%)	89% (71%-100%)	-	-
	2	38 (38%)	5 (13%)	97% (92%-100%)	91% (82%-100%)	83% (70%-98%)
	3	32 (32%)	5 (16%)	97% (91%-100%)	90% (80%-100%)	82% (69%-98%)
	4	6 (6%)	1 (17%)	80% (52%-100%)	-	-
	5	12 (12%)	7 (58%)	92% (77.3%-100%)	75% (54%-100%)	58% (36%-94%)
Primary tumor classification	pT2	46 (46%)	5 (11%)	98% (93%-100%)	92% (85%-100%)	89% (80%-100%)
	pT3	49 (49%)	15 (31%)	98% (94%-100%)	90% (82%-99%)	70% (57%-85%)
Post-PET sRT	No	65 (64%)	19 (29%)	94% (88%-100%)	84% (75%-94%)	71% (60%-85%)
	Yes	36 (36%)	2 (6%)	97% (92%-100%)	94% (87%-100%)	94% (87%-100%)

*CI* Confidence interval, *FFP* Freedom from progression, *PET* Positron emission tomography, *ISUP* International Society of Urological Pathology, *sRT* salvage radiotherapy

^a^No. (%)

On univariate analysis, there was no association between clinical progression at the last follow-up and the following factors: initial N stage; time from diagnosis to biochemical recurrence; PSA level, PSA doubling time (PSA-DT), and PSA velocity at time of PET/CT; and post-PET receipt of ADT. The initial pathological T stage (pT3 vs. pT2), ISUP grade (ISUP 5 vs. ISUP 1, 2, 3, and 4), and the receipt of post-PET sRT (yes vs. no) were significantly associated with clinical progression at the time of last follow-up. On multivariate analysis, initial ISUP grade 5 was significantly associated with clinical progression at the last follow-up. Furthermore, the use of sRT correlated significantly with lower clinical progression at the last follow-up. At the time of the last follow-up, all patients were alive; therefore, the overall survival (OS) was 100% (Table 3).

**Table 3:** Univariate and multivariate Cox regression analysis of clinical progression in patients with a prior negative PSMA PET/CT

Characteristics	Univariate analysis			Multivariate analysis		
	**HR**	**95% CI**	***P***^***£***^	**HR**	**95% CI**	***P***^***£***^
Initial T stage			**0.01**			
pT2	1.0	Reference		1.0	Reference	
pT3	3.8	1.4, 10.4	**0.01**	3.0	0.9, 10.0	0.06
Initial N stage			0.2			
pN0	1.0	Reference		-	-	-
pN1	2.3	0.7, 6.8	0.2	-	-	-
ISUP grade			**0.001**			
1	1.0	Reference		1.00	Reference	
2	0.9	0.2, 4.5	0.9	1.5	0.3, 9.0	0.6
3	0.9	0.2, 4.6	0.9	1.1	0.2, 5.8	0.9
4	1.1	0.1, 11.9	0.9	2.8	0.2, 36.2	0.4
5	5.5	1.1, 27.1	**0.03**	5.1	1.0, 26.1	**0.04**
Time from diagnosis to BCR	1.0	1.0, 1.1	0.8	-	-	-
PSA at PET/CT	1.3	0.3, 2.4	0.2	-	-	-
PSA-DT at PET/CT	1.0	0.9, 1.0	0.3	-	-	-
PSA Velocity at PET/CT	1.1	1.0, 1.2	0.1	-	-	-
Post-PET sRT			**0.01**			
No	1.0	Reference		1.00	Reference	
Yes	0.2	0.04, 0.83	**0.01**	0.20	0.04, 0.92	**0.03**
Post-PET ADT			0.4			
No	1.0	Reference		-	-	-
Yes	0.5	0.11, 2.16	0.4	-	-	-

*HR* hazard ratio, *CI* confidence interval, *BCR* biochemical recurrence, *ADT* androgen deprivation therapy, *PSA* prostate-specific antigen, *PSA-DT* prostate-specific antigen doubling time, *PET/CT* positron emission tomography/computed tomography, *ISUP* International Society of Urological Pathology, *RP* radical prostatectomy, *RT* radiation therapy, *sRT* salvage radiotherapy

^£^Based on Cox proportional hazards model

**Fig. 2:**
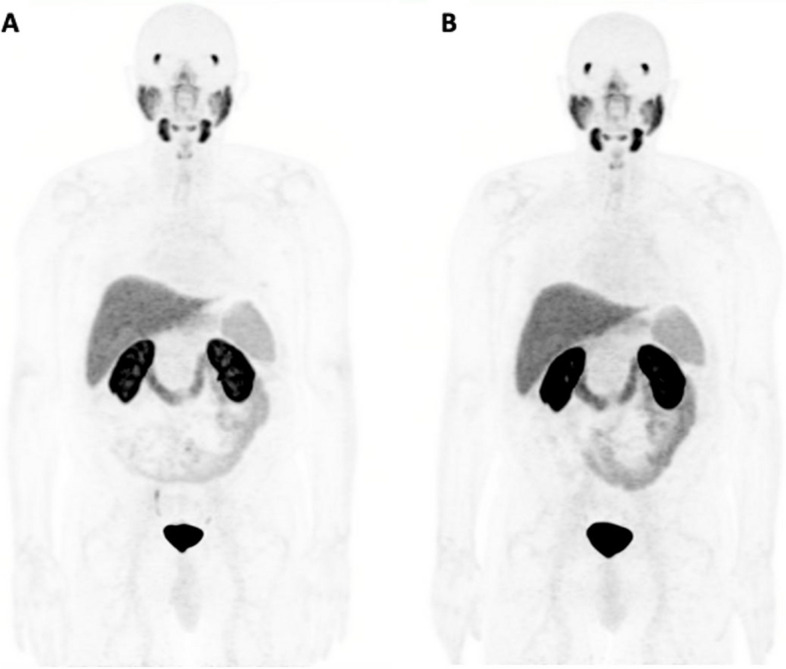
A 70-year-old patient with biochemical recurrence (PSA, 4 ng/mL; ISUP grade 3) after radical prostatectomy. Maximum Intensity Projection (MIP) of baseline [^18^F]DCFPyL PET/CT (**A**) shows tracer accumulation in the ureters but no evidence of clinical recurrence. The patient was followed by clinical/radiologic examinations. No recurrent prostate cancer was localized on repeat [^18^F]DCFPyL PET/CT scan after 25 months (PSA, 3.5 ng/mL) (**B**)

**Fig. 3:**
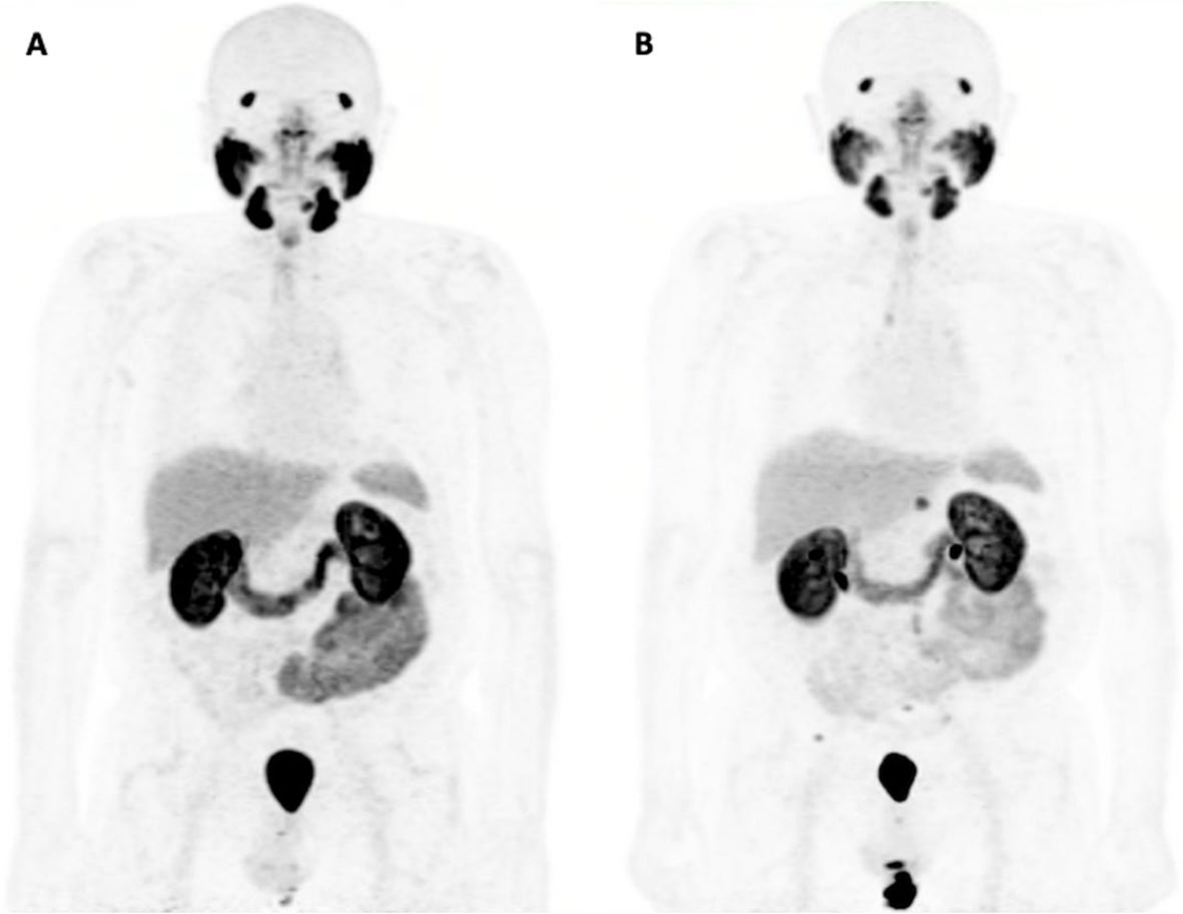
A 76-year-old patient with biochemical recurrence (PSA, 0.61 ng/mL; ISUP grade 5) after radical prostatectomy. Maximum Intensity Projection (MIP) of baseline [^18^F]DCFPyL PET/CT (**A**) shows no evidence of clinical recurrence. The patient was followed by clinical/radiologic examinations. Repeat [^18^F]DCFPyL PET/CT after 28 months (PSA, 8.78) (**B**) demonstrates multiple PSMA-avid lymph nodes involving the retrocrural, paraaortic, bilateral common iliac, left external iliac and mesenteric lymph nodes, as well as PSMA-avid osseous lesions involving the right acetabulum, T6 and T12 vertebral bodies

Representative baseline and follow-up [^18^F]DCFPyL PET/CT scans are illustrated in Figs. 2 and 3.

## Discussion

Early identification and localization of prostate cancer recurrence post-initial curative treatment are pivotal for refining patient management strategies. PSMA PET/CT imaging has significantly advanced our capability to detect both local recurrences and distant metastases in individuals with early biochemical recurrence of PCa, including those with low serum PSA levels where other imaging modalities fall short due to inadequate sensitivity. The beneficial impact of a positive PSMA PET/CT scan in directing treatment strategies for BCR patients towards achieving a biochemical response and potentially postponing clinically evident disease has been well-documented [29–32]. Yet, the role of a negative PSMA PET/CT scan in this scenario remains less clear, highlighting an unmet clinical need to determine its predictive value in patients with BCR. Specifically, it is important to determine if those patients benefit from early sRT, or whether a surveillance-based approach, potentially delaying salvage or systemic treatments and their associated comorbidities, could be considered.

Our study, with a median follow-up of 39 months involving 101 patients with biochemical recurrence of prostate cancer and negative [^18^F]DCFPyL PET/CT scans, has shed additional light on this issue. Among these patients, a significant portion was followed without further treatment, while a smaller group received sRT, either to the prostate bed alone or combined with pelvic lymph nodes. Our findings indicate that the median clinical progression-free survival (CPFS) was not reached for several subgroups. Kaplan–Meier analysis showed that the 3-year freedom from progression (FFP) rates were substantially and significantly higher in patients who underwent sRT compared to those who were followed without any active treatment. Multivariate Cox regression analysis pinpointed an initial ISUP grade 5 as significantly associated with clinical progression at the last follow-up. Moreover, receipt of sRT was significantly linked to lower clinical progression at the last follow-up, highlighting the clinical advantage of early sRT intervention in this patient group.

Despite advancements, the optimal timing and strategy for managing PSA-only recurrences continue to provoke debate. The presence of measurable PSA levels does not necessarily predict clinically evident metastatic disease. This underscores the importance of distinguishing between candidates for curative local treatments, applicable in cases of local recurrence or locoregional lymph node metastasis, and those for whom palliative care or stereotactic body radiation therapy is more suitable due to distant metastasis. This distinction underscores the critical role of advanced imaging modalities, like PSMA PET/CT, in accurately identifying the recurrence sites and disease extent, thereby guiding treatment decisions [33].

The introduction of PSMA PET/CT, capable of detecting recurrences at low PSA levels, has challenged traditional sRT protocols post-RP. This technique's precision in locating recurrences outside the prostate bed has introduced the potential for more targeted, image-guided sRT, offering a strategic shift from the early, less specific approach [34–36]. Delaying sRT until radiological evidence of recurrence raises concerns about its efficacy. However, this approach also has potential benefits, including the ability to target radiation therapy more accurately and reduce unnecessary treatments. Ongoing studies aim to compare PSMA-guided sRT with conventional sRT, focusing on survival and quality of life, with the goal of tailoring treatment strategies based on PSMA PET/CT findings [37]. Furthermore, integrating novel biomarkers and genomic classifiers promises to enhance postoperative risk assessments, potentially refining patient selection for sRT and systemic treatment adjustments [38–42].

A recent retrospective study involving 103 BCR patients with negative [^68^Ga]Ga-PSMA-11 PET/CT scans, who had not received any active treatment before clinical relapse, found that 55% experienced clinical relapse within a median follow-up of 22 months. Notably, clinical recurrences were predominantly located outside the prostate bed in 54% of cases. This study identified the primary PCa ISUP grade as a significant predictor of clinical relapse, with patients having ISUP grades 1 and 2 showing a notably longer clinical relapse-free survival compared to those with higher ISUP grades. This suggests that, for a subset of patients with less aggressive PCa experiencing biochemical recurrence at PSA levels below 0.5 ng/mL, a cautious approach might be warranted, potentially delaying unnecessary salvage radiation [24]. In another multicenter analysis by Emmett et al., [^68^Ga]Ga-PSMA-11 PET/CT scans were highly predictive of a 3-year freedom from progression, including a cohort of 29 patients with negative scans who were observed without immediate treatment. This group showed a continued PSA rise in 66% of cases, indicating that carefully selected patients with negative scans might be safely monitored without immediate intervention [25]. Scharl et al. also compared the outcome of PSMA PET guided sRT in 173 patients with negative PSMA PET scans against 168 with positive findings, revealing no significant difference in biochemical recurrence-free survival. This suggests the potential for early sRT use regardless of PSMA PET scan results [26]. Adebahr et al.'s retrospective multicenter study of 300 patients undergoing sRT post-BCR without PSMA PET evidence of disease showed that after a median 33-month follow-up, the three-year biochemical recurrence-free survival, metastatic-free survival, and overall survival rates post-sRT were promising. Multivariate analysis highlighted seminal vesicle infiltration, ISUP score above 2, and pre-sRT PSA levels as significant factors influencing outcomes, reinforcing the role of early sRT even in patients with negative PSMA PET scans [23]. This aligns with our findings, advocating for the strategic use of early sRT to optimize patient outcomes.

Previous research indicates that [^18^F]DCFPyL PET/CT has a negative predictive value of 82% and demonstrates high sensitivity in detecting disease within pelvic lymph nodes, extra-pelvic lymph nodes, bone, and visceral/soft tissue [43]. Despite these strengths, some patients with advanced disease might not be detected due to the limitations of PSMA PET/CT imaging. In response, recent studies have explored the differences in detection capabilities between PSMA PET scans and PET imaging using other radiotracers, such as 2-deoxy-2-[^18^F]fluoro-D-glucose (2-[^18^F]FDG) and ^18^F-sodium fluoride ([^18^F]NaF), to address this issue. Notably, the National Comprehensive Cancer Network (NCCN) does not currently endorse 2-[^18^F]FDG PET for staging or detecting prostate cancer recurrence. However, a systematic review by McGeorge et al. revealed that incorporating 2-[^18^F]FDG PET after PSMA PET could enhance metastasis detection rates in high-risk, early-stage castration-resistant prostate cancer patients from 65 to 73%, even when conventional imaging fails to reveal abnormalities. Remarkably, in 17% of cases with post-radical prostatectomy biochemical recurrence and negative PSMA PET scans, a subsequent positive 2-[^18^F]FDG PET scan was observed, suggesting a potential advantage in combining these molecular imaging approaches [44]. Furthermore, Harmon et al*.* observed significant discordance between [^18^F]NaF PET and PSMA PET in metastatic prostate cancer, indicating that PSMA expression and bone turnover might diminish in later stages of the disease. These observations imply that [^18^F]NaF PET/CT could provide additional insights into the presence of bone metastases in patients with negative PSMA PET/CT scans [45]. Therefore, considering alternate molecular imaging in certain clinical scenarios might be beneficial, though further investigation is needed to evaluate the clinical and economic implications of employing multiple imaging modalities to minimize false negatives.

In our analysis, pelvic lymph nodal and prostatic fossa recurrences emerged as the primary sites of clinical recurrence among patients with BCR and negative PSMA PET/CT scans. This observation aligns with the growing interest in incorporating pelvic nodal radiation alongside prostate bed treatment. The RTOG 0534 (SPPORT) trial, which included patients with either consistent or initially undetectable and subsequently rising PSA levels post-RP, explored the outcomes of administering RT solely to the prostate bed, combined with short-term ADT, or targeting both the pelvic lymph nodes and prostate bed with short-term ADT. This study did not find significant OS differences across the groups. However, it noted improved disease progression freedom with the combined pelvic node and prostate bed RT plus short-term ADT approach [46]. Our study did not have sufficient power to detect a significant difference in freedom from progression between patients receiving sRT to just the prostatic fossa and those treated at both sites. Given the current evidence, including pelvic nodes in the RT field for all BCR patients in the PSMA PET era is not yet a standard practice, particularly without clear OS benefits as seen in the SPPORT trial. Identifying patients at greater risk for pelvic node metastasis post-RP, who might benefit from elective pelvic irradiation alongside prostate bed treatment, remains a key challenge.

Patients with negative PSMA PET/CT benefiting from sRT suggests that prostate bed recurrence may have been underdiagnosed by PSMA PET imaging. Some PSMA radiotracers, notably [^18^F]DCFPyL and [^68^Ga]Ga-PSMA-11, are excreted by the kidneys with high accumulation in the bladder, which may not be completely overcome by administration of diuretics such as furosemide [47]. This may mask localized recurrences near the bladder neck. More lipophilic radiotracers excreted predominantly by hepatobiliary clearance have been proposed as alternatives [48], since these compounds have lower activity in the bladder. Others have recommended the use of longer-lived radioisotopes such as ^89^Zr, which enable delayed imaging after complete bladder emptying [49]. Very early imaging at 5 min post-injection may also improve the detection of local recurrence [50]. Alternatively, low volume disease or lesions expressing low quantities of PSMA protein might cause false negative PSMA PET/CT examinations.

The present study is subject to limitations inherent to its retrospective analyses, including the potential for unequal risk factor distribution and selection bias. The relatively small cohort of 101 patients limits the statistical power of our findings. Additionally, the diversity in diagnostic imaging modalities used to detect clinical relapse introduces variability into our analysis. A notable imbalance exists between the number of patients who underwent sRT and those managed conservatively without further treatment. Furthermore, our study did not investigate treatment-related toxicities, although we observed no toxicities requiring medical intervention or hospitalization throughout the follow-up period. Another limitation of our study is the significant difference in time from diagnosis to recurrence between the active surveillance and sRT groups, with median times of 7 years and 2 years, respectively. This disparity suggests that patients with shorter times to recurrence may have been more likely to receive sRT. This could have influenced treatment selection and, consequently, the observed treatment outcomes. Patients with more rapidly recurring disease might be considered for more aggressive treatment approaches, including sRT, to manage their recurrence more effectively. This factor should be considered when interpreting the efficacy of sRT observed in our study. Additionally, the median follow-up time of 36 months for the sRT group may be considered short for evaluating long-term outcomes. There is also presumably some selection bias in determining which patients received ADT. Patients with more aggressive disease might have been more likely to receive ADT, potentially skewing the results towards more favorable outcomes in this group. Furthermore, the type and duration of ADT varied among patients, ranging from 6 to 24 months, with the majority receiving 12 months of therapy. While the analysis did not show a significant impact of ADT on the main outcome variable, this variability in ADT administration could affect individual outcomes and should be considered when interpreting the results.

## Conclusions

In conclusion, patients with biochemical recurrence after radical prostatectomy who have a negative PSMA PET/CT scan have superior freedom from progression when treated by salvage radiotherapy compared to patients undergoing surveillance only. The analysis also underscores the prognostic significance of the initial ISUP grade, with ISUP grade 5 being associated with worse outcomes. These findings lend support to the consideration of early sRT as a viable intervention for patients with negative PSMA PET findings, emphasizing the importance of tailoring treatment approaches based on both PSMA PET results and tumor-specific characteristics. Conducting further research, particularly randomized controlled trials, remains important to validate these findings and optimize treatment protocols for this patient population.

## Data Availability

The data presented in this study are available on request from the corresponding author. The data are not publicly available due to ethical considerations.
